# Urethral injury in penile fracture: a narrative review

**DOI:** 10.1590/S1677-5538.IBJU.2020.99.02

**Published:** 2020-01-10

**Authors:** Rodrigo Barros, José Genilson Alves Ribeiro, Heleno Augusto Moreira da Silva, Flávio Rondinelli de Sá, Angelo Maurilio Fosse, Luciano A. Favorito

**Affiliations:** 1 Departamento de Urologia Universidade Federal Fluminense NiteróiRJ Brasil Departamento de Urologia, Universidade Federal Fluminense – UFF, Niterói, RJ, Brasil;; 2 Unidade de Pesquisa Urogenital Universidade Estadual do Rio de Janeiro Rio de JaneiroRJ Brasil Unidade de Pesquisa Urogenital, Universidade Estadual do Rio de Janeiro – UERJ, Rio de Janeiro, RJ, Brasil

**Keywords:** Penis, Urethra, Urethral Stricture

## Abstract

**Objective:**

To present the evolution and the recent data on the etiology, diagnosis, management and outcomes of penile fracture (PF) with concomitant urethral injury.

**Materials and Methods:**

We searched the Pubmed database between 1998 and 2019 using the following key words: “penile fracture”, “fracture of penis”, “trauma to penis”, “rupture of corpora cavernosa”, “urethral injury”, “urethral rupture” and “urethral reconstruction”.

**Results:**

The incidence of urethral lesion in patients with PF varies by geographic region and etiology. Blood in the meatus, hematuria and voiding symptoms are highly indicative of urethral rupture. The diagnosis of PF is eminently clinical and complementary exams are not necessary. The treatment consists of urethral reconstruction and the most common complications found are urethral stenosis and urethrocutaneous fistula.

**Conclusion:**

PF is an uncommon urological emergency, particularly in cases with urethral involvement. Urethral injury should be suspected in the presence of suggestive clinical signs, and diagnosis is usually clinical. Urgent urethral reconstruction is mandatory and produces satisfactory results with low levels of complications.

## INTRODUCTION

Penile fracture (PF) with associated urethral rupture is an extremely rare condition. The urethral lesion can be partial or complete and the incidence varies from 1% to 38%, depending the geographic region and etiology (1, 2).

Patients usually report a cracking sound with concomitant sudden swelling and ecchymosis of the penis followed by immediate detumescence. Blood in the meatus, hematuria and urinary retention may be experienced with urethral injury (3). Studies have variously reported the usefulness of retrograde urethrocystography (RGU), ultrasound (USG), flexible cystoscopy and magnetic resonance imaging (MRI) in the diagnosis (4-7). PF and urethral injury should be treated by surgery with the goal of preserving sexual potency and regaining normal micturition function (8, 9).

It is important to address these issues in the urological literature. Therefore, in this review, we present the evolution and the recent data on the etiology, diagnosis, management and outcomes of PF with concomitant urethral injury.

## MATERIALS AND METHODS

We searched the Pubmed database between 1998 and 2019 using the following key words: “penile fracture”, “fracture of penis”, “trauma to penis”, “rupture of corpora cavernosa”, “urethral injury”, “urethral rupture” and “urethral reconstruction”. Special emphasis was given to relevant articles reporting the etiology, management and outcomes of PF with associated urethral rupture. All English papers were included and non-English papers were excluded.

## DISCUSSION

The incidence of urethral lesion in patients with PF was reported to be only 3% in Eastern European countries, Asia, and Africa, where the main cause was penile manipulation. In an Iranian study with 352 cases of PF, the main cause was the practice of *taqaandan* in 269 cases (76.4%). This is a self-inﬂicted injury, consisting of intentional forceful acute bending of part of the shaft of the erect penis in a downward, upward, or lateral direction while holding the other part stationary, to achieve detumescence of the penis, as a practice to release tension, among other reasons. In this series, there was combined penile and urethral rupture only in ﬁve cases (10). Et Atat et al. described their experience with 300 cases of PF, with masturbation as etiology in 180 (60%) cases. Concomitant urethral injury was found in only five (1.6%) patients, corroborating the theory that non-coital injury has a lower incidence of urethral involvement due to low-energy trauma (11).

**Figure 1:**
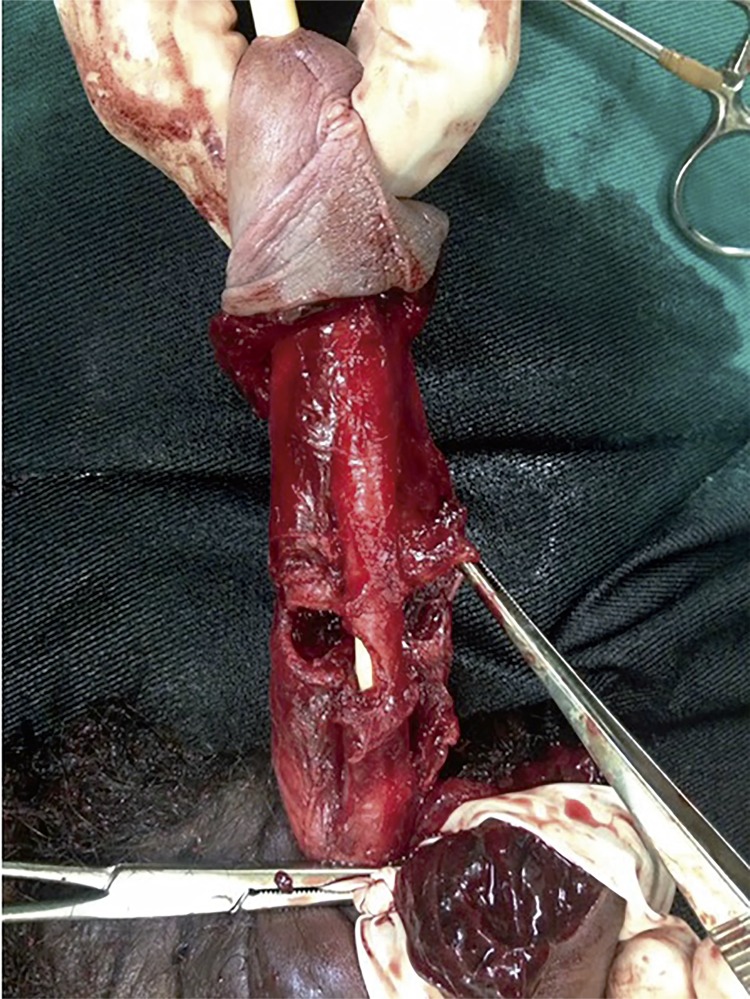
The figure shows a penile fracture with urethral injury and bilateral rupture of the corpora cavernosa.

On the other hand, the incidence reached 38% in western countries where sexual intercourse represented the main cause of PF (12). The incidence of urethral injury was higher in these countries, such as Brazil and the United States, because intercourse is generally associated with high-energy traumas. Nason et al. reported a retrospective analysis of 21 PF cases in Ireland and all fractures were the result of sexual misadventure (13). A Brazilian study evaluated the relationship between sexual position and severity of PF in 90 patients. According to the results, the positions with the “man on top” and “doggy style” were considered the most severe, presenting greater association with urethral and bilateral lesions of the corpora cavernosa (14) Figure-1.

**Figure 2:**
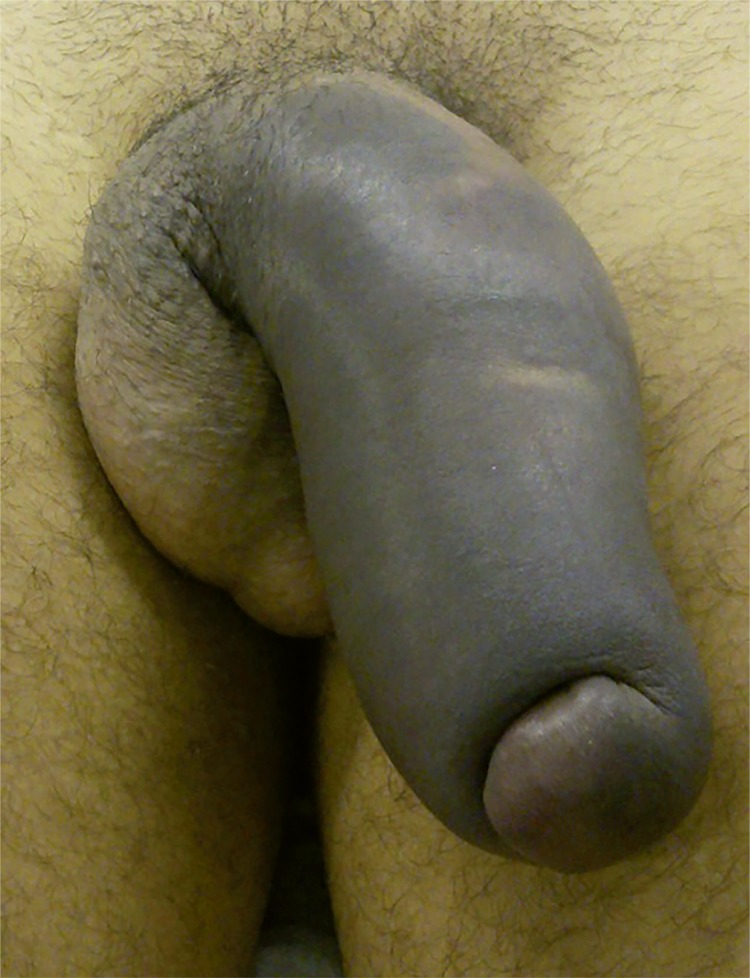
Tipical eggplant aspect in a patient with penile fracture and urethral injury.

Penile fracture generally causes a cracking sound followed byrapid detumescence, sudden swelling and ecchymosis of the penis, so that itacquires an aspect known as “eggplant deformity” (3) Figure-2. Blood in the meatus, hematuria and voiding symptoms are highly indicative of urethral rupture, but the absence of these findings does not exclude urethral lesions (15). A recently published systematic review found that 50% of cases of urethral injury were clinically asymptomatic and the lesion was found accidentally during USG or intraoperatively (16).

**Figure 3:**
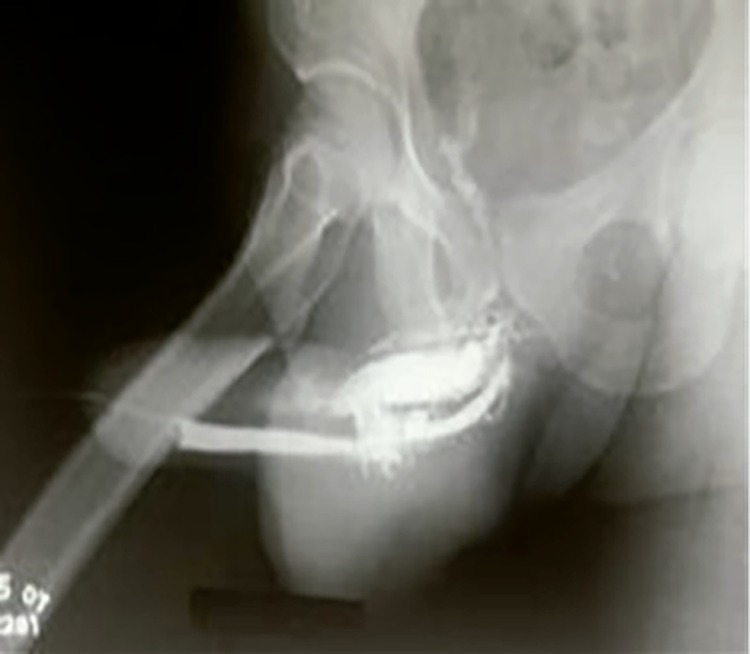
The figure shows a uretrocistography of a patient with penile fracture and urethral injury.

In suspected cases of urethral injury, RGU may demonstrate contrast leakage at the lesion site and reveal the exact point of urethral injury (17) Figure-3. Some authors consider RGU to be compulsory if diagnosis of urethral rupture is suspected (18). However, RGU can show false negative results in up to 28.5% of cases (17). Therefore, there is no consensus on the role of RGU in PF (15). Also, trying to assess the possibility of concomitant urethral injury, Kamdar et al. described the use of flexible cystoscopy at the same time as surgical repair, allowing direct visualization of the urethra without delaying treatment. However, not all emergency hospitals have a flexible cystoscope (5). Although the site of injury had 100% correlation with intraoperative findings, color Doppler ultrasound can miss urethral rupture (6). MRI is highly associated with intraoperative findings of tunical rupture, presenting 100% sensitivity and 77.8% specificity. On the other hand, MRI has lower accuracy for urethral lesions, with 60% sensitivity and 78.3% specificity (7). Therefore, the diagnosis of PF is eminently clinical and these complementary exams are not necessary, especially when urethral lesion is suspected and surgical intervention is always required (19).

The objective oftreating PF with associated urethral injury is to preserve sexual potency and recover normal micturition function. The treatment consists of tension-free end-to-end anastomosis under a transurethral catheter. A circular subcoronal incision followed by further penile degloving is the best described surgical approach, allowing good exposure of the corpus cavernosum and urethra, besidesidentification and repair of any concomitant urethral injury (9).The corpus cavernosum is treated using interrupted 3-0 polyglactin sutures. Partial urethral tearing is primarily treated with simple 5-0 polyglactin sutures over an 18 French catheter. In cases of complete urethral injury, the treatment consists of tension-free end-to-end anastomosis after sufficient dissection of the urethra on both sides of the tear (8, 19). The postoperativeduration of urethral catheterization depends on the complexity of observed lesions. Generally, the urethral catheter is left for 10-14 days in cases of partial injury and for 14-21 days in cases of complete lesion (8). Some authors recommend suprapubic cystostomy in cases of complete circumferential rupture. They believe that it is safer to place a suprapubic catheter and and recommend keeping it closed for at least 3 days after urethral catheter removal to ensure adequate and normal voiding before its removal (4).

The main tools described in the literature to assess postoperative urinary function are the International Prostate Symptom Score (IPSS) questionnaire and uroflowmetry. While the IPSS questionnaire is subjective, uroflowmetry is a very objective way to determine urinary flow and screen for possible abnormalities. Some studies have observed urinary deterioration using IPSS in around 30% of patients with PF after urethral reconstruction (8, 12).

El-Assmy et al. used uroflowmetry in patients with urethral injury after surgical treatment of PF and found abnormal urinary flow due to urethral stenosis in only one case (20, 21). Raheem et al. observed similar results and only one of ten patients had abnormal flow (4). RGU is recommended when abnormalities are found in the IPSS questionnaire or uroflowmetry to identify possible urethral stricture or other complications. (8, 22-24). Short penile urethral stenosis can be treated with sequential dilatations (4, 21). Another complication is urethrocutaneous fistula. Usually patients experience deterioration in urinary function according to the IPSS questionnaire analysis and the diagnosis is confirmed through RGU. Small fistulas can be treated conservatively with a urethral catheter for around 30 days (8). Some authors have suggested using grafting to interpose the suture to avoid fistulous trajectory formation. A subcutaneous abscess may occur in patients with a full urethral lesion who underwent end-to-end urethroplasty due to small extravasation of urine between the points, causing collection of urine, despite the use of the urethral catheter. This can be treated with percutaneous drainage and oral antibiotic therapy with a satisfactory outcome (8). Di Pierro et al. reported a case of urethral pseudodiverticulum after urethral injury in PF and management of the case conservatively with cystostomy for two months after surgery (22).

Although the treatment of urethral injury in PF is of interest to the urological community, we found in the literature review a number of quality case reports and small single institution case series, with few studies composed of larger series or providing details regarding follow-up and voiding function after surgery (Table-1).

**Table 1:** – Findings of urethral injury in penile fracture and outcomes from selected series.

Study	Total Pf	Confirmed Urehtral Injury N (%)	Urethral Injury Cases Include On The Study	Urethral Bleeding	Partial/ Total Injury	Coital Etiology N	Preoperative Imaging Used	Treatment	Follow-Up N	Complications
Deiruche 2008	312	10 (3.2)	10	10	10/0	4	None	Primary urethroplasty	10	None
Ibrahim 2010	155	14 (9)	14	13	11/3	7	RGU was performedin three patients	Primary urethroplasty	12	One case of relative narrowing in the penile urethra
Raheem 2014	246	34 (13.8)	34	34	22/12	11	RGU was performed in all patients	Primary urethroplasty + Suprapubic catheter#	12+	One case of ring stricture in the anterior urethra
Barros 2018	175	27 (15.4)	13	10	9/4	13	None	Primary urethroplasty	13	One case of urethrocutaneous fistula and another of subcutaneous abscess

**#** = Suprapubic catheterinsertion or not was determined by the surgeon’s preference. **+** = Only in cases of complete urethral disruption.

## CONCLUSIONS

Penile fracture is an uncommon urological emergency, particularly in cases with urethral involvement. Urethral injury should be suspected in the presence of suggestive clinical signs, such as urethral bleeding, hematuria or urinary retention, and in cases with bilateral cavernosal rupture. Diagnosis is usually clinical and complementary diagnostic methods are not required. Urgent urethral reconstruction is mandatory and produces satisfactory results with low levels of complications.
